# Mechanistic Elucidation of BBOX-Catalyzed Hydroxylation and THP-Induced Oxidative Rearrangement via QM/MM Calculations

**DOI:** 10.3390/molecules31111941

**Published:** 2026-06-03

**Authors:** Zheng Ruan, Hong Li, Yongjun Liu, Xianghui Zhang, Xinyi Li

**Affiliations:** 1 Shandong Huameng Traditional Chinese Medicine Research Co., Ltd., Jinan 250002, China; 2 College of Pharmacy, Shandong University of Traditional Chinese Medicine, Jinan 250355, China; 3 School of Chemistry and Chemical Engineering, Shandong University, Jinan 250100, China; 4 Institute of Chinese Materia Medica Chemistry, Shandong Academy of Chinese Medicine, Jinan 250013, China

**Keywords:** γ-butyrobetaine hydroxylase (BBOX), α-ketoglutarate-dependent dioxygenase, QM/MM method, oxidative rearrangement mechanism, carnitine biosynthesis

## Abstract

Carnitine plays an essential role in fatty acid metabolism, and its biosynthesis is tightly regulated by γ-butyrobetaine hydroxylase (BBOX), an Fe(II)/α-ketoglutarate-dependent dioxygenase. BBOX is the target of mildronate (THP), a clinically used drug for treating ischemic heart diseases. However, the detailed mechanisms of BBOX-catalyzed hydroxylation and the atypical oxidative rearrangement underlying THP inhibition remain elusive. In this study, we employed combined quantum mechanics/molecular mechanics (QM/MM) methods to systematically elucidate these mechanisms at the atomic level. Our calculations reveal that the hydroxylation of γBB proceeds via a classical three-step mechanism in the quintet state, with hydrogen atom abstraction as the rate-determining step. Remarkably, substitution of the C4 methylene group in γBB with an amino group in THP redirects the reaction pathway, as the lone pair electrons on the adjacent nitrogen atom render N-N bond cleavage kinetically favored over hydroxyl rebound, thereby blocking carnitine synthesis. Through systematic evaluation of possible rearrangement pathways, we rule out the previously proposed direct 1,2-H migration and suggest a revised mechanism featuring imine-mediated hydrogen transfer, hydroxyl rebound preceding C-C bond formation, and final radical coupling. This work provides a detailed atomic-level understanding of both the catalytic and inhibitory mechanisms of BBOX, revealing how substrate electronic effects dictate reaction outcomes. The elucidated mechanistic insights offer a theoretical foundation for understanding the catalytic versatility of the αKG-dependent dioxygenase family and provide valuable guidance for the rational design of novel BBOX inhibitors.

## 1. Introduction

**Figure 1:**
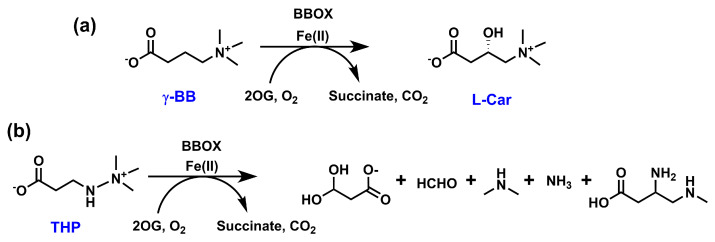
(**a**) The hydroxylation of γBB catalyzed by BBOX. (**b**) The inhibition reaction of BBOX when THP is the substrate.

Carnitine (L-3-hydroxy-4-N,N,N-trimethylaminobutyrate) plays a central role in energy metabolism in eukaryotic cells. Its primary function is to serve as an essential carrier that facilitates the transport of long-chain fatty acids from the cytosol into the mitochondrial matrix for β-oxidation, a process that is critical for maintaining energy homeostasis in tissues highly dependent on fatty acid oxidation, such as the heart and skeletal muscle [1,2]. In mammals, carnitine is derived from both dietary intake and endogenous biosynthesis [3]. The latter originates from Nε-trimethyllysine (TML) and proceeds through four enzymatic steps [4], with the final and rate-limiting step being catalyzed by γ-butyrobetaine hydroxylase (BBOX) [5] (Figure 1a). BBOX belongs to the Fe(II)/α-ketoglutarate (αKG)-dependent dioxygenase superfamily [6,7,8,9,10]. Members of this superfamily catalyze diverse oxidative transformations via a high-valent Fe^IV^=O intermediate, which is widely recognized as the key oxidant in these systems. The geometric and electronic structures of this intermediate, particularly its high-spin (S = 2) ground state and frontier molecular orbital (FMO) composition, have been the subject of intense experimental and computational scrutiny by Bollinger, Krebs, Solomon, and Neese [11,12,13]. These studies have suggested that the reactivity of the ferryl species is critically dependent on the orientation of the substrate relative to the Fe=O bond and the nature of the equatorial ligands [14]. Fe(II)/α-ketoglutarate-dependent dioxygenases play essential roles in diverse physiological processes, such as epigenetic regulation, collagen biosynthesis, and hypoxia sensing [15].

Given the pivotal role of carnitine in lipid metabolism, perturbations in its metabolic flux are closely associated with various diseases [16,17]. On the one hand, elevated levels of carnitine can be metabolized by the gut microbiota to generate trimethylamine N-oxide (TMAO), which has been strongly correlated with an increased risk of atherosclerosis and cardiovascular diseases [18,19]. On the other hand, under pathological conditions such as myocardial ischemia, excessive reliance on fatty acid oxidation leads to increased oxygen consumption and the accumulation of toxic metabolic intermediates [20]. Accordingly, pharmacological intervention aimed at moderately suppressing endogenous carnitine biosynthesis has emerged as a potential therapeutic strategy for ischemic heart diseases, including myocardial infarction [21,22]. Mildronate (3-(2,2,2-trimethylhydrazinium) propionate, THP), a competitive inhibitor of BBOX, is clinically used for the treatment of angina pectoris and myocardial infarction [23,24]. Its pharmacological action mainly arises from inhibition of endogenous carnitine biosynthesis. This inhibition reduces fatty acid β-oxidation and shifts myocardial energy metabolism toward the more oxygen-efficient glucose oxidation pathway under ischemic conditions, while simultaneously decreasing TMAO production [25]. Therefore, elucidating the inhibitory mechanism of THP toward BBOX is of great significance for understanding its cardioprotective effects and for the rational development of novel metabolic modulators [26].

A detailed understanding of the structure and function of BBOX provides a fundamental basis for the rational design of its inhibitors. Crystal structures of BBOX in complex with its natural substrate γ-butyrobetaine (γBB) and the inhibitor THP have been determined by the Schofield group (PDB: 3O2G, 3MS5) [27]. Structural analyses reveal that BBOX exists as a homodimer with a catalytic C-terminal double-stranded β-helix (DSBH) domain characteristic of Fe(II)/αKG-dependent dioxygenases [28]. The active site contains a characteristic ″2-His-1-Asp″ iron-binding motif composed of His202, Asp204, and His347. Compared with analogous non-heme Fe/αKG dioxygenases such as AlkB and TauD, BBOX exhibits a rigid active site microenvironment that restricts substrate conformational rearrangement [29,30]. Structural comparisons further demonstrate that BBOX shares a high degree of sequence and structural homology with Nε-trimethyllysine hydroxylase (TMLH), the enzyme responsible for catalyzing the first step of carnitine biosynthesis, together constituting a distinct subclass within this enzyme family [31,32].

**Figure 2:**
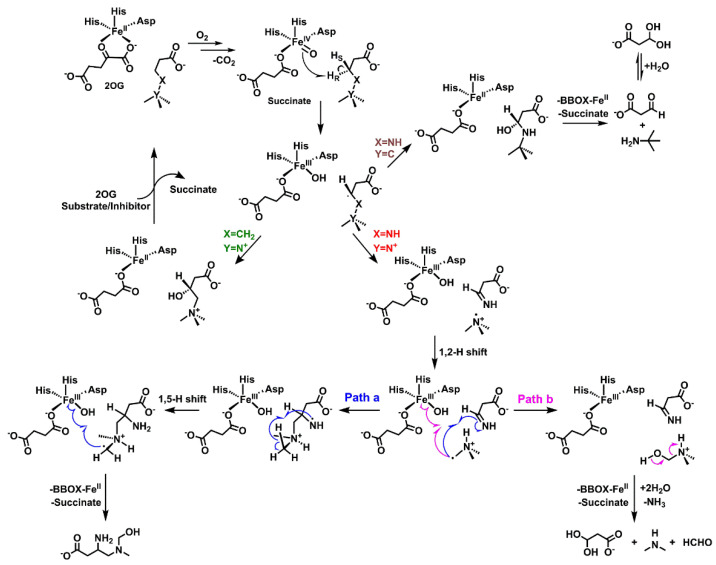
Proposed mechanisms for the reaction of γBB (green), THP (red), and 3-tert-butylamino propionic acid (brown) catalyzed by BBOX. The blue and pink arrows indicate different pathways leading to distinct products when THP serves as the substrate.

Experimental investigations into the mechanism of THP action have revealed the complexity of BBOX catalysis. The binding mode of THP within the active site closely resembles that of γBB [27]. In particular, the positively charged quaternary ammonium/hydrazinium head groups of both ligands are accommodated within an aromatic cage formed by aromatic residues such as Tyr and Trp, where cation–π interactions play a key role in substrate recognition and positioning [33]. However, it has been found that when THP acts as a substrate, BBOX does not catalyze the canonical hydroxylation reaction. Instead, an unusual N-demethylation coupled with oxidative rearrangement occurs, producing products such as formaldehyde, dimethylamine, malondialdehyde semialdehyde, and 3-amino-4-(methylamino)butanoic acid (AMBA) (Figure 1b). Based on these observations, a radical-based mechanistic hypothesis has been proposed (Figure 2). Initially, the Fe^IV^=O intermediate abstracts a hydrogen atom from the C3 position of THP, generating a carbon-centered radical. The absence of Me_3_N^+^NH_2_ in experimental detection suggests that N-N bond cleavage is kinetically favored over hydroxyl rebound, highlighting the critical role of the nitrogen substituent in determining the reaction pathway. Subsequent radical transformations have been proposed to involve hydrogen transfer and rearrangement steps leading to the formation of a new C–C bond, ultimately producing AMBA. This transformation has been suggested to resemble a Stevens-type rearrangement, although it proceeds under remarkably mild enzymatic conditions compared with the harsh conditions typically required in organic synthesis [34,35,36].

Although this mechanistic framework provides an important basis for understanding the inhibitory action of THP, the detailed reaction mechanism remains elusive due to the transient nature of the intermediates and the limitations of current experimental techniques. In particular, the sequence of hydrogen transfer events, the timing of hydroxyl rebound, and the precise mechanism of C–C bond formation remain unclear.

To address these unresolved mechanistic questions, we systematically characterized the reaction mechanism of BBOX-catalyzed hydroxylation of γBB leading to L-carnitine formation. We then investigated the THP-induced oxidative rearrangement pathway in detail. Specifically, this work aims to address the following key questions: (1) What is the complete reaction pathway, energetic profile, and spin-state selectivity governing BBOX-catalyzed hydroxylation of the native substrate γBB? (2) What is the fundamental origin of the drastic change in reaction pathway upon substitution of γBB with THP? (3) What is the detailed mechanism underlying the oxidative rearrangement of THP?

By addressing these questions, the present study provides an atomic-level mechanistic framework for understanding the catalytic versatility of α-ketoglutarate-dependent dioxygenases and offers insights that may guide the rational design of BBOX-targeted therapeutics [37].

## 2. Results

### 2.1. Mechanisms of BBOX-Catalyzed Hydroxylation of γBB to L-Carnitine

**Figure 3:**
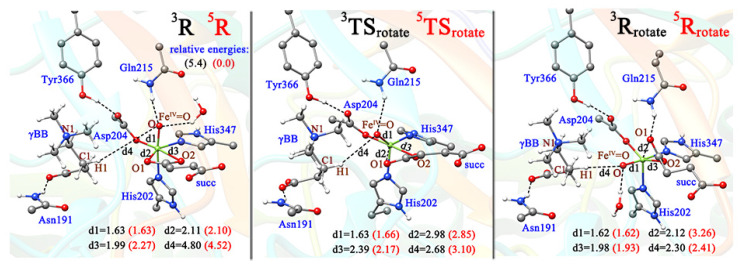
Optimized structures involved in the isomerization of Fe^IV^=O with the γBB as the substrate. All relative energies are given in kcal/mol and distances are in Ångström.

Substantial progress has been made in elucidating the early stages of catalysis in αKG-dependent oxygenases, particularly regarding O_2_ binding and the formation of the high-valent iron–oxo intermediate [38,39,40,41,42,43]. In the generally accepted mechanism [29,44,45,46,47,48,49], molecular oxygen enters the active site and coordinates to the Fe center at the position initially occupied by a water molecule, accompanied by its displacement. The bound O_2_ then rapidly reacts with αKG, leading to the oxidative decarboxylation that produces succinate and CO_2_, and ultimately generates the highly reactive Fe^IV^=O intermediate. Based on this, the calculations in this work do not focus on this process, but rather begin with the reaction of the Fe^IV^=O intermediate. Therefore, the reactant structure R of the Fe^IV^=O complex was first optimized in the triplet and quintet states (Figure 3).

To verify the robustness and reliability of the initial structural model that was constructed on the snapshot at 30 ns, we additionally selected four additional representative snapshots extracted from the MD trajectory at 15, 17, 20, and 25 ns, respectively. All these alternative conformations were subjected to full QM/MM geometry optimization for the reactant complex at the quintet. Structural comparison demonstrated that both the overall protein backbone and the active site of the BBOX, including the orientation of the Fe^IV^=O center and the substrate, key bond parameters and hydrogen-bonding network, were in excellent agreement with that obtained from the 30 ns snapshot (Figure S1). In addition, the representative structure extracted from the 500 ns MD simulation was also subjected to QM/MM optimization for the quintet reactant and named ^5^R_0_. The comparison of ^5^R_0_ with ^5^R is shown in Figure S2. It can be seen that the coordination of the active sites, the orientation of Fe^IV^=O, the binding mode of the substrate, and the hydrogen bond network are highly similar. Therefore, the highly consistent structural features among these multiple independent snapshots confirm that the initial structure used in this work is structurally representative and stable, which further validates the rationality of the constructed computational model and guarantees the reliability of the calculated energy profiles and mechanistic results.

From an energetic perspective, the quintet state is the ground state, lying 5.4 kcal/mol lower than the triplet state in relative energy. The quintet spin preference and Fe(IV)=O bonding property of the BBOX ferryl intermediate align with the established electronic structure for ferryl species in enzymes, which favors high-spin configurations due to weak-field ligands and is crucial for efficient HAT, which further verifies our computational results [12,50,51,52]. Structural analysis of the reactant complex shows that in both spin states, the distances between the Fe^IV^=O moiety and the *pro*-*R* hydrogen (H1) at the C1 position are relatively long (4.85 and 4.64 Å for the triplet and quintet states, respectively), which is unfavorable for the subsequent hydrogen atom abstraction (HAA) step. Therefore, the Fe^IV^=O intermediate is expected to undergo a conformational isomerization to reorient toward the target H1 atom of γBB. This geometric rearrangement is consistent with the mechanistic paradigm observed in other non-heme iron enzymes, where the ferryl oxygen must attain a specific geometric alignment (often described as “in-line” or “off-line”) relative to the target C-H bond to facilitate efficient H-atom abstraction [53]. The calculated energy barrier for this isomerization is comparable to that observed in the halogenase SyrB2, where a similar reorientation is required for selective halogenation [54]. To probe this process, a potential energy scan was performed along the dihedral rotation of the Fe^IV^=O unit in the enzyme environment. For the quintet state, the isomerization proceeds with an energy barrier of 12.9 kcal/mol, and the resulting rotated conformer (^5^R_rotate_) is 4.3 kcal/mol more stable than the initial reactant state (^5^R), indicating that this isomerization is both kinetically and thermodynamically feasible.

In the isomerized ^5^R_rotate_ structure, the Fe^IV^=O⋯H1 distance is reduced to 2.41 Å, and the relative orientation is well suited for efficient hydrogen atom abstraction (Figure 3). Concomitant with the repositioning of the oxo group, the succinate ligand undergoes a dihedral rotation along the Fe–O axis, such that one of its carboxylate groups occupies the original position of the oxo ligand, while maintaining bidentate coordination to the iron center throughout the process. During this isomerization, the hydrogen bond between Gln215 and the Fe^IV^=O moiety is disrupted. Instead, Gln215 forms a new hydrogen bond with the O1 atom of the succinate carboxylate group, while the Fe^IV^=O unit is stabilized by hydrogen bonding interactions with a nearby water molecule.

**Figure 4:**
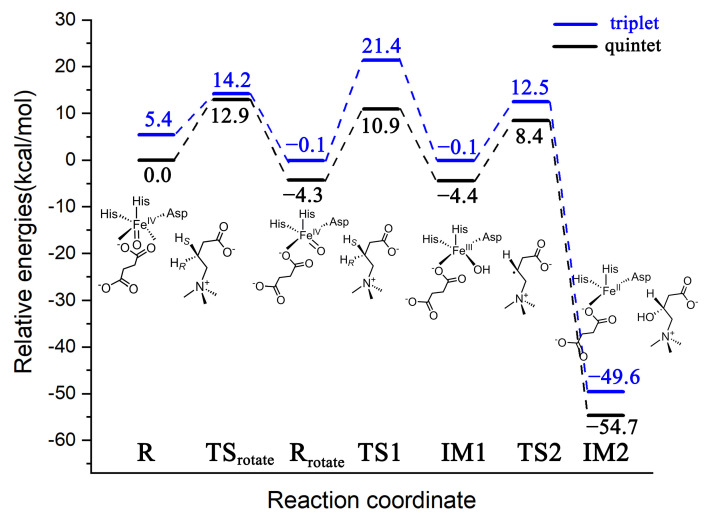
Energy profile for the BBOX-catalyzed hydroxylation of γBB at triplet and quintet states.

Following isomerization, the Fe^IV^=O intermediate abstracts the nearest H1 atom from γBB, which is subsequently followed by hydroxyl rebound to complete the hydroxylation process and yield L-carnitine (Figure S3). On the quintet spin surface, the calculated energy barriers for the HAA and hydroxyl rebound steps are 15.2 and 12.8 kcal/mol, respectively (Figure 4). These values are consistent with the previously reported barriers for other Fe/2OG enzymes. For instance, For example, the typical HAT energy barrier values for taurine dioxygenase (TauD) and syringomycin biosynthesis enzyme 2 (SyrB2), as well as other related nonheme iron enzymes, reported by Shaik and de Visser et al., are in the range of 14–18 kcal/mol [44,54,55,56,57,58]. In the ethylene-forming enzyme (EFE), which catalyzes both hydroxylation and ethylene formation, the HAT barrier for the L-Arg hydroxylation pathway was computed to be 15.7 kcal/mol [57]. Our computed value for BBOX falls within this range, suggesting a comparable intrinsic reactivity of the ferryl intermediate in activating aliphatic C-H bonds.

Furthermore, we also investigated the abstraction of *pro-S* hydrogen (H2) of C1 by Fe^IV^=O and found that the energy barrier was 36.5 kcal/mol at the quintet state, which is much higher than that for the abstraction of *pro-R* hydrogen (Figure S4). In addition, supplementary test calculations were performed by expanding the original QM region to incorporate Arg360, which forms a stable salt bridge with the succinate group in the BBOX active site. We optimized and compared the structures and relative energies of key transition states and intermediates along the reaction pathway from the initial reactant ^5^R to the hydrogen abstraction intermediate ^5^IM1, as well as ^3^R (Figure S5). The computational results demonstrate that inclusion of the Arg360–succinate salt bridge only induces minor deviations in local structural parameters and energetic differences. No obvious changes were observed in conformational structures, and the energy barriers of subsequent reaction steps. These additional test calculations confirm that the salt bridge interaction exerts no substantial influence on the overall reaction mechanism and energetic profile, thereby validating the rationality and robustness of our original QM region partition and computational model. All structural and energetic details are provided in Figure S5. Our results indicate that the final hydroxylation step in BBOX-catalyzed L-carnitine biosynthesis proceeds through three sequential stages: (i) isomerization of the Fe^IV^=O intermediate, (ii) hydrogen atom abstraction from the *pro*-*R* hydrogen at the C1 position of the substrate, and (iii) hydroxyl rebound. Among these steps, hydrogen atom abstraction constitutes the rate-determining step, and the overall reaction is more favorable on the quintet spin surface.

### 2.2. Inhibition Mechanism When THP Is Used as a Substrate

Considering that THP can competitively bind to BBOX in place of γBB, thereby suppressing endogenous carnitine biosynthesis and leading to reduced intracellular carnitine levels, which has significant physiological and pathological implications, we subsequently investigated the inhibition mechanism of BBOX when THP acts as a substrate.

#### 2.2.1. Competing Pathways of N-N Bond Cleavage and Hydroxylation

**Figure 5:**
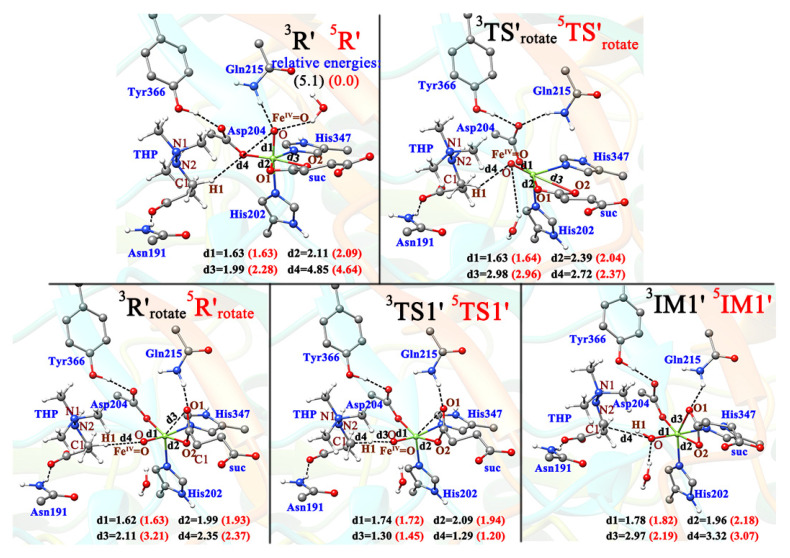
Optimized structures involved in isomerization of Fe^IV^=O and hydrogen abstraction. All distances are given in Ångström and relative energies are in kcal/mol.

**Figure 6:**
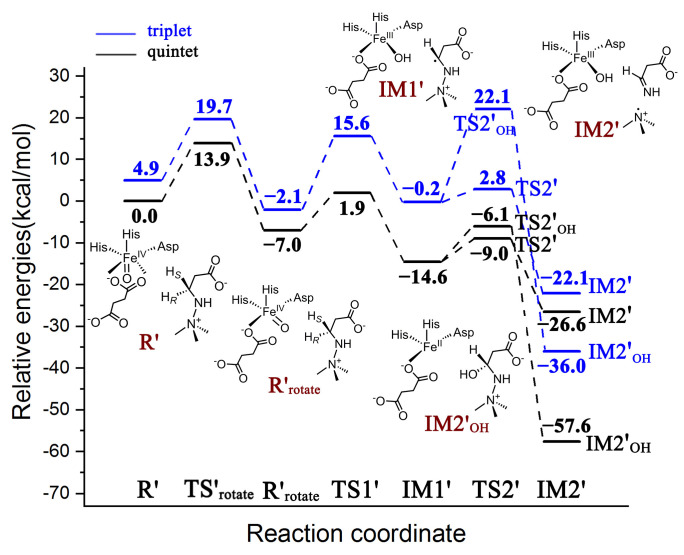
Energy profile of the isomerization of Fe^IV^=O, hydrogen abstraction, N-N bond cleavage and hydroxylation when THP serves as the substrate in the triplet and quintet states.

Based on the substrate structure of the crystals, it is known that THP and γBB have almost identical relative positions at the active sites. Therefore, the C atom in R mentioned above was replaced with a N atom, and QM/MM optimization was performed, as shown in Figure 5. It can be seen that the hydrogen bonding situation around the substrate THP, the Fe^IV^=O complex, and succinic acid in R′ is similar to that in R. The quintet state remains the ground state, and the relative energy of the triplet state is 5.1 kcal/mol higher than that of the quintet state. The distances between O and H1 in the triplet and quintet states are 4.85 and 4.64 Å, respectively. Therefore, to carry out the hydrogen abstraction reaction at the C1 position of the substrate, R′ would first undergo the isomerization of Fe^IV^=O. Calculations show that this process is similar to the reaction in R, with an energy barrier of 13.9 kcal/mol (Figure 6), producing R′_rotate_, where the distance between H1 and Fe^IV^=O is 2.37 Å.

Owing to the relatively short reaction distance, hydrogen abstraction at C1 in R′_rotate_ occurs more readily, with an energy barrier of 8.9 kcal/mol in the quintet, generating the substrate carbon radical intermediate ^5^IM1′ (Figure 5 and Figure 6). In ^5^IM1′, the distance between C1⋯O is 3.07 Å, suggesting that either hydroxyl rebound to form a hydroxylated product or N-N bond cleavage may occur.

Both possible pathways were calculated. The results indicate that the hydroxyl rebound process is associated with an energy barrier of 20.7 kcal/mol (Figure 6), which remains accessible at ambient temperature. In contrast, N-N bond cleavage is significantly more favorable, requiring only 5.6 kcal/mol in the quintet state (Figure S6 and Figure 5), thereby supporting the N-N bond cleavage mechanism proposed in previous experimental studies [27]. By comparing the reactions of substrate γBB and THP, it is speculated that the difference in reaction pathways may be due to the different electronic properties of N and C. The previous hydrogen abstraction step generates a carbon radical intermediate. Since N has lone pairs of electrons, the N-N bond is easier to break, thus generating an N=N double bond. However, the carbon atom does not have lone pairs of electrons, so after the hydrogen abstraction, hydroxyl rebound is more likely to occur. Notably, the high barrier of OH rebound for THP also originates from the rigid binding mode confined by cation–π interactions in the active-site aromatic cage, which restricts substrate conformational rearrangement and results in unfavorable spatial distance and orientation between the carbon radical and Fe–OH group. And the high intrinsic OH rebound barrier also agrees with the mechanistic view that unfavorable substrate radical–Fe(OH) orbital overlap will significantly elevate rebound energy [14].

#### 2.2.2. 1,2-H Migration Reaction

**Figure 7:**
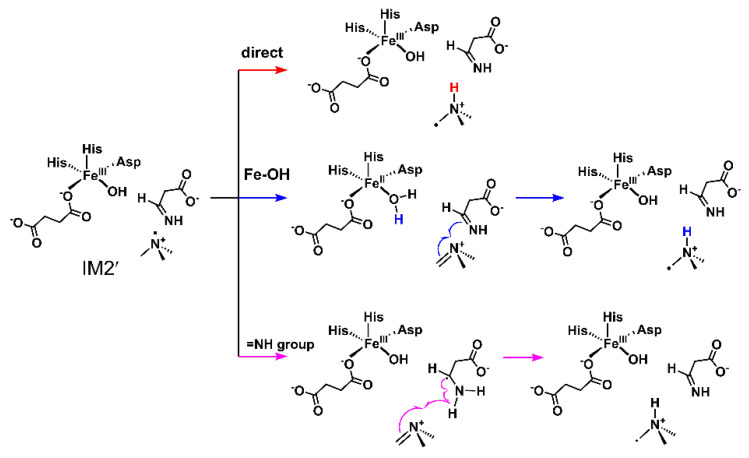
Proposed three pathways of 1,2-H migration in this work. The red arrow represents the direct intramolecular 1,2-H migration pathway, the blue arrows represent the 1,2-H migration pathway via the Fe-OH center, and the pink arrows represent the 1,2-H migration pathway mediated by imine.

Following the breakage of the N-N bond, intramolecular 1,2-H migration may occur, generating a methylene radical. Based on the optimization results of the reactants and calculations of processes such as hydroxylation and N-N bond formation, the quintet state is the ground state of the reaction. Therefore, calculations for subsequent reactions were performed in the quintet state. In IM2′, N1 is attached to three equivalent methyl groups, with the nearest H atom at a distance of 2.08 Å from N1, making it the most likely candidate for 1,2-H migration. However, calculations show that direct intramolecular 1,2-H migration is difficult, with an energy barrier exceeding 45 kcal/mol. Since this step does not involve material and electron transfer with the metal center, a simple model was constructed in aqueous solution, and QM calculations were performed for this step. The results are largely consistent with those obtained in the enzyme, with an energy barrier of 47.2 kcal/mol, thus confirming that direct transfer of H from the trimethylammonium methyl group to N is not easy. Therefore, other possible pathways were explored (Figure 7). Observing the N(CH_3_)_3_^+^ region near the BBOX active site, it can be found that the imine generated after N-N bond cleavage and the Fe-OH center may act as proton acceptors to assist in the transfer of H atoms. The abstraction of the nearest methyl hydrogen atom from IM2′ by Fe-OH was first investigated. Due to the high reactivity of the Fe-OH active species, hydrogen abstraction by Fe-OH can proceed, generating water molecules coordinated with iron (Figure S7). However, the cleavage of the O-H bond in the water molecule is relatively difficult. Therefore, the possibility of Fe-OH acting as a proton relay station was ruled out through calculation.

Subsequently, the possibility of the imine acting as a proton-transfer relay was investigated. As shown by IM2′ in Figure S6, the N1⋯N2 distance is 2.45 Å, and the methyl groups at C2 and C3 are both in close proximity to the Fe–OH moiety, making them potential candidates for 1,2-H migration. The transfer of H2 to the N2 atom was examined through potential energy surface scanning and subsequent optimization (IM2′_H2_ → TS3′_H2_ → IM3′_H2_, Figure S7c). The calculations indicate that this process is endergonic, with the resulting IM3′_H2_ intermediate lying 10.8 kcal/mol higher in energy than IM2′_H2_. Furthermore, back-transfer of H2 to N1 is associated with an additional energy increase, suggesting that the resulting methylene intermediate is intrinsically unstable. Overall, the 1,2-H migration mediated by the imine relay involves a total energy barrier of 12.4 kcal/mol.

#### 2.2.3. Oxidative Rearrangement Mechanism

Experimental identification of the products indicates that following N-N bond cleavage, the resulting intermediate would undergo two successive hydrogen-transfer events from the N1-methyl groups to generate methylene intermediates. One of the resulting methylene radicals subsequently forms a bond with the imine carbon, whereas the other undergoes hydroxyl rebound to yield a hydroxylated intermediate. Consequently, the sequence of 1,2-H migration, C-C bond formation, and hydroxyl rebound remains uncertain, implying multiple plausible mechanisms.

**Figure 8:**
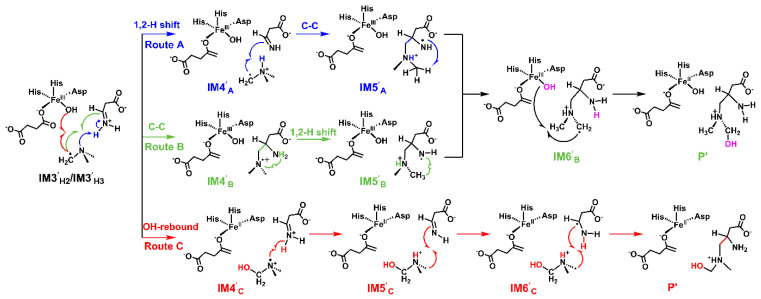
Three proposed pathways for IM3′ to P′. Route A (blue) represents the pathway in which 1,2-H migration occurs first, followed by the formation of C-C bond, and then 1,5-H migration and hydroxyl rebound. Route B (green) represents the pathway in which C-C bond formation precedes 1,2-H migration. Route C (red) represents the pathway in which OH rebound occurs preferentially.

On this basis, three possible reaction pathways were proposed (Figure 8). Route A follows the mechanism suggested in previous studies, in which 1,2-H migration occurs first, and the resulting methylene radical attacks the C=N bond of the imine to form a C-C bond, followed by 1,5-H migration and subsequent hydroxyl rebound to afford the final product. In contrast, Routes B and C represent alternative mechanisms, in which C-C bond formation or hydroxyl rebound occurs after the formation of the first methylene intermediate and is followed by a 1,5-H migration step, ultimately yielding the product.

Accordingly, the three proposed pathways mentioned above were investigated in detail. For route A, analysis of IM4′_A_ shows that the orientation and proximity of the methylene C1 atom are favorable for C–C bond formation with C3. However, our calculation results showed that as C1 approaches C2, the H2 atom readily transfers back to N2, preventing optimization of the corresponding C-C bond formation intermediate. This observation is consistent with the energetic analysis described above. The formation of the IM4′_H2_ intermediate is endergonic, and the trimethylammonium radical cation is intrinsically unstable. As a result, the hydrogen atom on N1 is highly labile (Figure S8). Consequently, as C1 and C2 approach each other, the cleavage of the N1-H2 bond is readily induced. These findings effectively rule out the feasibility of this pathway previously proposed in the literature.

In light of the intrinsic instability of the IM4 intermediate, an alternative mechanism was proposed in which C-C bond formation precedes H2 rebound (route B in Figure 8). Given that C1 is a radical with limited nucleophilic character, formation of the C-C bond requires overcoming a moderate energy barrier of approximately 19.7 kcal/mol. Along this process, the C1⋯C2 distance decreases from 3.64 Å in IM3′_H2_ to 1.40 Å at TS4′_B_, ultimately yielding a C1-C2 bond length of 1.68 Å in IM4′_B_ (Figure S9).

Spin density analysis indicates that the spin population on N1 is 0.68, identifying N1 as the primary radical center. Moreover, because C1 is bonded to an ammonium cation moiety, the resulting C1-C2 bond is elongated relative to a typical C-C single bond. Following C-C bond formation, the rebound of the H2 atom to N1 becomes more favorable, proceeding with an energy barrier of 10.5 kcal/mol and leading to the formation of IM5′_B_, which is thermodynamically stable. Upon transfer of the H2 atom, a significant redistribution of spin density is observed, with the spin population on N1 decreasing to 0.01, while that on N2 increases to 0.87, indicating migration of the radical center from N1 to N2.

Structural analysis of the IM5′_B_ intermediate suggests that to generate a methylene radical, there is no suitable nearby residue capable of acting as a proton acceptor. Under these conditions, a 1,5-H shift appears to be the more plausible pathway. In this mechanism, N2 serves as the most likely proton acceptor, and the hydrogen atom on C3 is the closest to N2 at this stage. Calculations show that this 1,5-H migration is associated with an energy barrier of 23.5 kcal/mol and is also endergonic, leading to the formation of the imine intermediate IM6′_B_ (Figure S9).

Subsequent hydroxyl rebound would be required to yield the final product. However, in IM6′_B_, the distance between the C3-centered radical and the Fe–OH moiety is as large as 6.23 Å. Based on previous studies of hydroxyl rebound in αKG-dependent non-heme iron enzymes, such an extended separation renders the rebound step highly unfavorable. Therefore, route B is also unlikely to occur.

**Figure 9:**
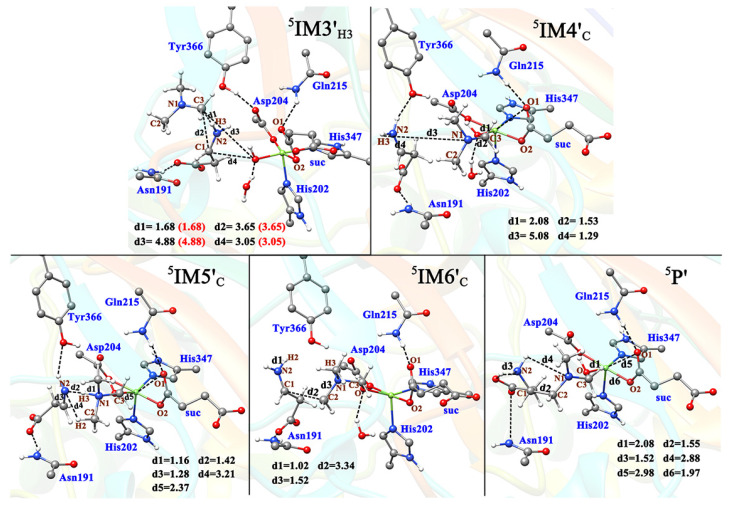
Optimized structures involved in the transfer of H3 atom, hydroxylation and C-C bond formation processes. All distances are given in Ångström.

Given the relatively distant C3⋯O atom in IM6′_B_ along route B, it is speculated that the OH rebound process may occur preferentially, as shown in route C in Figure 8. Structural analysis of ^5^IM2′ indicates that among the three methyl groups attached to N1, only the C3 methyl group is in close proximity to the Fe-OH moiety and is suitably positioned to serve as the site for hydroxyl rebound. Accordingly, the reaction pathway was further explored starting from ^5^IM2′, where the H3 atom transfers to N2, followed by hydroxyl rebound from the Fe-coordinated OH group to the C3-centered radical. The H3 transfer to N2 is found to be relatively facile, with a low energy barrier of only 5.6 kcal/mol. Notably, during the scanning of the hydroxyl rebound process, it was observed that, in contrast to other αKG-dependent enzymes, the displacement of the OH group is minimal, and it consistently maintains coordination with the Fe center (Figure 9).

In most previously reported enzyme-catalyzed hydroxyl rebound processes, the substrates are typically bulky or possess extended aliphatic chains. In addition, they are often stabilized at the active site through hydrogen-bonding interactions between their polar functional groups and nearby polar amino acid residues. As a consequence, during hydroxyl rebound, it is more likely that the smaller OH radical undergoes substantial displacement to form a bond with the substrate-centered radical. In contrast, the hydroxylated species in IM3′_H3_ is the N(CH_3_)_3_^+^ moiety, which is relatively small and lacks stabilizing interactions with surrounding residues. This allows it to move more freely within the active site.

**Figure 10:**
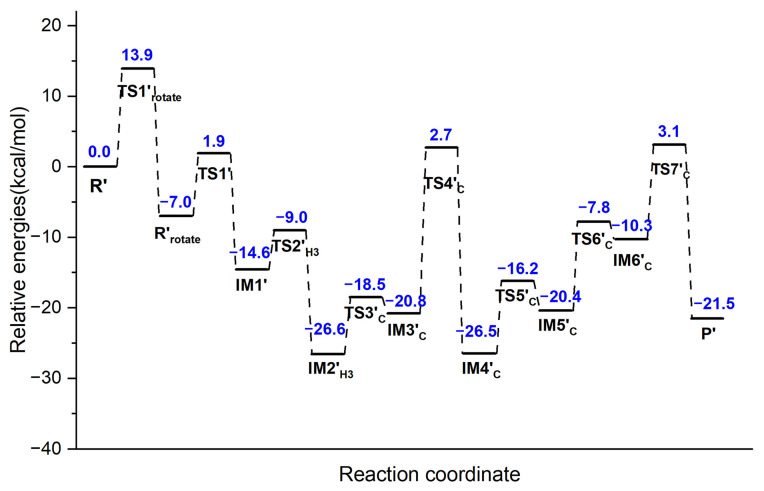
Energy profile for the catalytic reaction of BBOX when THP serves as the substrate.

Moreover, its proximity to the Fe–OH unit facilitates maintenance of the six-coordinate geometry at the iron center, thereby contributing to overall energetic stabilization of the system. The transformation from IM3′_H3_ to IM4′_C_ involves an electron transfer event, in which the iron center is reduced from Fe(III) to Fe(II). Subsequently, IM4′_C_ undergoes H3 transfer from N2 back to N1, regenerating an imine intermediate. This is followed by the transfer of the H2 atom from the C2 methyl group to N2, generating a C2-centered radical while concomitantly cleaving the C1=N2 double bond to form a C1-centered radical. Finally, radical coupling between C1 and C2 leads to the formation of the C1-C2 bond, ultimately yielding the oxidatively rearranged product (Figure 10).

## 3. Discussion

In this study, we employed a combined QM/MM approach to systematically elucidate the complete catalytic mechanism of BBOX-catalyzed hydroxylation of its native substrate γBB and the unprecedented oxidative rearrangement induced by the inhibitor THP. Our computational results not only corroborate previous experimental observations but also significantly improve and deepen the understanding of the complex inhibition mechanism of BBOX.

The calculated mechanism for the canonical hydroxylation of γBB aligns with the well-established mechanism for FeII/αKG-dependent dioxygenases [55,59,60]. The reaction proceeds through three sequential steps: isomerization of the high-valent Fe^IV^=O intermediate, HAA from the *pro*-*R* hydrogen at the C1 position of γBB, and subsequent hydroxyl rebound. The quintet state is identified as the ground state, consistent with the high-spin nature of the Fe^IV^=O species commonly observed in this enzyme family. The HAA step, with a calculated barrier of 15.2 kcal/mol on the quintet surface, serves as the rate-determining step. This energy barrier is in qualitative agreement with the experimentally determined turnover number for BBOX, underscoring the reliability of our computational model.

A pivotal finding of this study is the mechanistic basis for the dramatic shift in reactivity upon substitution of γBB with THP. The only structural difference between the two molecules is the replacement of a methylene group (C4) in γBB with an amino group in THP. This seemingly minor change profoundly alters the catalytic pathways. Our calculations reveal that after the initial HAA step, the resulting carbon-centered radical intermediate IM1′ is at a critical branch point. Due to the presence of the adjacent nitrogen atom with its lone pair of electrons, the N-N bond cleavage is kinetically far more favorable, with an energy barrier of only 5.6 kcal/mol. In contrast, the competing hydroxyl rebound pathway faces a significantly higher barrier of 20.7 kcal/mol. This provides a clear and direct explanation for why THP acts as an inhibitor rather than a conventional substrate: the electronic properties of the heteroatom effectively hijack the normal catalytic cycle, diverting it towards an alternative, non-productive pathway. This finding highlights a fundamental principle in enzyme catalysis where subtle changes in substrate electronics can dictate the accessibility of distinct chemical transformations, expanding the catalytic functions of this enzyme.

**Figure 11:**
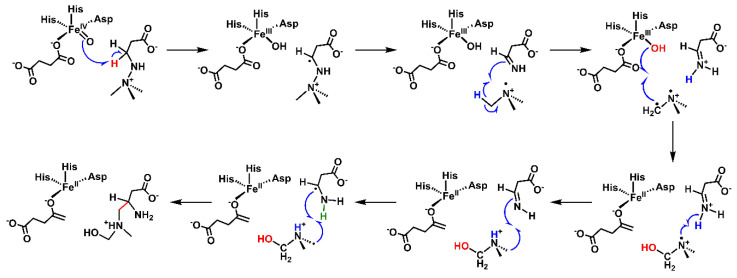
Proposed inhibition mechanism of BBOX using THP as the substrate based on the calculations in this work.

Furthermore, our work provides a critical re-evaluation and correction of the subsequent oxidative rearrangement mechanism proposed for THP (Figure 11). The previously hypothesized direct 1,2-H migration from the trimethylammonium group was found to be energetically unfeasible, with a calculated barrier exceeding 45 kcal/mol. Through systematic exploration of multiple pathways, we propose a revised and energetically viable mechanism (route C). This mechanism is characterized by several key features. First, the imine nitrogen acts as a proton relay, facilitating the transfer of hydrogen atoms with moderate energy barriers. Importantly, the hydroxyl rebound occurs prior to C-C bond formation. This specific order is rationalized by the spatial arrangement within the active site, where the small N(CH_3_)_3_^+^ moiety can move to maintain six-coordinate geometry around the iron center during the rebound step, thereby stabilizing the system. In addition, the rearrangement concludes with a radical coupling step, forming a new C-C bond. This calculated mechanism resolves the ambiguities in previous proposals and provides a complete atomic-level picture of the rearrangement, where the overall rate-determining step is identified as the hydroxyl rebound (23.5 kcal/mol). Notably, our observation of a stepwise radical process involving electron transfer (ET) and subsequent rearrangement mirrors the mechanism recently elucidated by Shaik et al. for the Fe/2OG desaturase DfmD [14]. In both cases, the enzyme machinery suppresses the thermodynamically favored hydroxylation (or other side reactions) by orchestrating a geometrically controlled radical rebound or cyclization. This highlights a strategy among Fe/2OG enzymes to divert the reactive radical intermediate toward diverse functionalization outcomes.

In addition, the intermediate IM2′_H3_ is energetically ~5 kcal/mol lower than the final product on the quintet energy profile (Figure 10), demonstrating that IM2′_H3_ is thermodynamically more stable. This stability originates from effective radical delocalization across the N–N hydrazinium framework in IM2′_H3_, which affords prominent resonance stabilization. In contrast, the final oxidative rearrangement product possesses a newly constructed C–C coupled skeleton with subtle structural and angle strain confined by the BBOX active-site microenvironment, which inevitably increases the overall energy of the product. Despite the higher thermodynamic stability of IM2′_H3_, the subsequent hydrogen migration, hydroxyl rebound and radical coupling processes remain kinetically accessible under enzymatic conditions. The protein active-site environment can modulate the reaction energetics and drive the reaction progression toward the final product, even starting from a thermodynamically favored intermediate state.

The mechanistic characteristics and energetic results of BBOX in this work show clear consistencies and subtle differences with previously reported nonheme Fe/2OG oxygenases from the collected literature. First, the rigid substrate binding mode in BBOX leads to poor radical–cofactor orientation, which is similar to the rebound suppression mechanism in SyrB2 and VioC summarized by Bollinger, Krebs and Solomon groups [58,61,62,63]. The stabilization of intermediates in THP systems arises from N-atom lone-pair delocalization in the BBOX substrate, analogous to the arginine-based 2OG enzyme systems [61]. Moreover, we ruled out the previously proposed direct 1,2-H migration mechanism with an ultrahigh energy barrier (>45 kcal/mol), which is consistent with the conclusion in the latest BBOX computational study that direct 1,2-H shift is kinetically infeasible [64]. Instead, our revised pathway featuring imine-mediated hydrogen transfer, prior hydroxyl rebound, and final radical C–C coupling is energetically more favorable, and shares similar stepwise radical rearrangement characteristics with DfmD and other Fe/2OG-dependent oxidative rearrangement enzymes [43]. In addition, similar to the bifunctional 2OG enzymes studied by Christov, Cisneros and de Visser et al., BBOX also presents obvious pathway branching regulated by substrate electronic effect and active-site microenvironment, which further enriches the mechanistic understanding of nonheme iron dioxygenases [65,66]. Furthermore, the calculated energy profile for THP diverges dramatically from that of γBB, demonstrating how a seemingly minor change in substrate structure, such as the introduction of a nitrogen heterocycle with a lone pair capable of interacting with the metal center, can radically redirect the catalytic pathways. This echoes mechanistic lessons from other divergent enzymes, such as VioC [50], where substrate chirality dictates reaction outcome through precise positioning in the active site, and AspH, where an unusual second-sphere water network plays a critical role in catalysis [49].

The structural and mechanistic insights gained from this study have significant implications. From a biological perspective, the THP-induced reaction not only blocks carnitine synthesis by consuming the co-substrates but also generates reactive intermediates, amplifying its inhibitory effect. The discovery that BBOX can catalyze such a complex rearrangement expands the known reaction scope of the αKG-dependent dioxygenase family, suggesting that these enzymes may possess greater synthetic potential than previously recognized. From a drug design perspective, our findings provide a theoretical foundation for developing novel BBOX inhibitors. Strategies to enhance the binding interactions of quaternary ammonium groups within the aromatic cage, or the introduction of substituents that facilitate N-N bond breaking or stabilize key radical intermediates, hold promise for developing more effective and selective therapeutics. Our constructed energy profiles, particularly the identification of the rate-limiting step, offer valuable insights for the kinetic evaluation of future inhibitor candidates.

## 4. Models and Methods

### 4.1. Construction of the Initial Model and Molecular Dynamics Simulations

The initial structure of the enzyme-substrate complex was constructed based on the crystal structure of BBOX containing γBB (PDB ID: 3O2G) [27]. Previous studies on Fe(II)/αKG-dependent oxygenases have shown that the O atoms of the high-valence ferro-oxygen complex formed after αKG oxidation are basically located at the O_2_ binding site. Based on this crystal structure, the zinc atom was replaced with the iron atom to construct a Fe^IV^=O complex at the coordinated water molecule, and the αKG analog NOG was manually modified to decarboxylated succinic acid. The iron atom has a six-coordinate configuration, and the ligands include the oxygen atom, His202, His347, Asp204 and succinic acid. In addition, the crystal structure is a homodimer containing two identical single chains, with the active site located inside the spatial structure of the enzyme protein. Therefore, considering the computational efficiency, only one chain was retained for modeling and MD simulations.

### 4.2. Molecular Dynamics Simulations

Hydrogen atoms were added to the entire system after carefully assigning the protonation states of titratable residues. These protonation states were determined based on p*K*_a_ values predicted by PROPKA [67] in combination with inspection of the local hydrogen-bonding environment. All Arg and Lys residues were treated as protonated, whereas all Glu and Asp residues were modeled in their deprotonated forms. For the histidine residues, His15, His82, His136, His202, and His347 were protonated at the δ position, while His179, His209, His210, His218, and His275 were protonated at the ε position.

After adding hydrogen atoms, the positions of the hydrogen atoms were first optimized using the CHARMM22/CMAP all-atom force field [68] implemented in the CHARMM program [69] through the 200 steps of steepest descent followed by 200 steps of Newton–Raphson minimization. The system was then solvated using a spherical water shell with a radius of 38 Å, and three Na^+^ ions were randomly introduced to ensure overall charge neutrality. The resulting pre-equilibrated model comprised 24,028 atoms, including 6528 TIP3P water molecules. Prior to MD simulations, the solvated system underwent a series of energy minimizations. Subsequently, a 100 ns MD simulation was performed at 300 K and 1 bar under stochastic boundary conditions using the CHARMM22/CMAP all-atom force field to equilibrate the system. During the MD simulations, the Fe^IV^=O moiety and the residues in the first coordination shell were kept fixed. The simulation results indicate that the system reached equilibrium after approximately 7 ns (Figure S10), and a snapshot of the trajectory at 30 ns was extracted for subsequent QM/MM calculations.

To further verify the rationality and conformational stability of the enzyme-substrate complex structure, we additionally performed a 500 ns long-time MD simulation using the GPU-accelerated Amber version 22 package [70]. The force field parameters for the iron ion were generated using the MCPB.py module implemented in AmberTools version 23 [71,72]. The substrate was described with the general AMBER force field (GAFF) [73], while the protein residues were parameterized using the Amber ff19SB force field [74]. Partial atomic charges were obtained via the RESP method at the B3LYP/def2-TZVP level of theory [75]. The whole system was solvated explicitly in a rectangular TIP3P water box under periodic boundary conditions, maintaining a minimum distance of 10 Å between the protein surface and the box boundary. To achieve overall charge neutrality, two Na^+^ ions were placed around the protein surface.

The RMSD plot of the protein backbone during the MD simulation is presented in Figure S11, which shows that the system gradually reaches equilibrium and maintains stable fluctuation after 150 ns, indicating that the overall structure of the enzyme-substrate complex is well converged without large conformational changes. The statistical distribution of the O-H1 distance in the active site throughout the 500 ns trajectory is summarized in Figure S12, and the average O-H1 distance is maintained at approximately 4.3 Å, which is consistent with the structural characteristics of the representative snapshot obtained by MD simulation using the CHARMM program. Through K-means clustering analysis of all conformations in the trajectory, the representative structure for QM/MM calculations was selected as the most frequent conformational state, accounting for 21.6% of the total trajectory.

### 4.3. QM/MM Calculations

**Figure 12:**
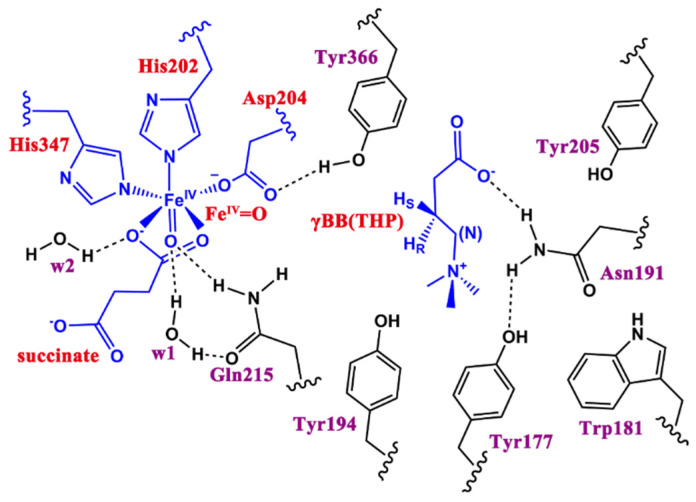
Selected QM region in this work.

By comparing the crystal structures of BBOX-bound γBB (PDB ID: 3O2G) and THP (PDB ID: 3MS5) [27], it was found that the binding position of THP in BBOX is almost identical to that of γBB. Therefore, in the construction of the QM/MM model containing the inhibitor THP, γBB in the crystal structure was manually modified to THP; that is, the C-4 methylene group was replaced with an amino group, and optimization was performed for subsequent QM/MM calculations. The entire system was divided into QM and MM regions. The QM region includes the substrate (γBB or THP), the Fe^IV^=O complex, Fe-coordinated residues (His347, His202, Asp204) and succinic acid. In addition, residue Gln215 forms a hydrogen bond with Fe^IV^=O, affecting the binding of O_2_. The carboxyl group of γBB is located by forming hydrogen bonds with the side chain of Asn191 and the main chain of Tyr205. The N-trimethylammonium group of positively charged γBB is located in an electron-rich aromatic “cage” formed by the side chains of Tyr177, Tyr194 and Trp181, and Tyr366 also has hydrogen bond interactions with the side chain of Asp204. Therefore, the above residues are also included in the QM region to ensure the reliability of the calculation results (Figure 12).

All QM/MM calculations were performed using the ChemShell package version 3.3.2 [76] in conjunction with TURBOMOLE version 7.1 [77] and DL_POLY version 2.20 [78]. An electron intercalation scheme was used to explain the polarization effect of the enzyme environment on the QM region. Hydrogen-bonded atoms with charge-displacement models were used to handle the QM/MM boundaries. Calculations for the QM region were performed using density functional theory, and for the MM region, the CHARMM force field was used. For geometry optimization, the LANL2DZ ECP basis set was used for iron atoms, and the 6-31G (d, p) basis set was used for all other atoms [79]. Single-point energy calculations were further corrected using larger basis sets: the LANL2TZf basis set was used for iron atoms, and the 6-311++G (2d, 2p) basis set was used for other atoms. This computational scheme is commonly utilized and well benchmarked in mechanistic simulations of non-heme 2-oxoglutarate-dependent iron enzymes and high-valent Fe^IV^=O intermediates [80,81,82,83]. All transition states (TS) were first identified as maxima on the potential energy surface (PES) along the reaction coordinates, and then fully optimized using the P-RFO optimizer [84] in the HDLC code [85]. To further validate the stationary points located on the potential energy surface, vibrational frequency calculations were performed for all key transition states at the same level as the optimization calculations. Each transition state exhibited one imaginary frequency corresponding to the expected reaction coordinates. Owing to the large size of the QM/MM model and the high computational cost associated with full Hessian calculations, frequency analyses were restricted to the QM region. The obtained vibrational modes confirmed the nature of the optimized transition states. The corresponding imaginary frequencies are summarized in Table S3. Furthermore, empirical dispersion correction was performed using the DFT-D3 program [86].

## 5. Conclusions

In summary, this study employed a QM/MM approach to systematically investigate the catalytic mechanism of BBOX for the hydroxylation of its native substrate γBB and the inhibitory oxidative rearrangement induced by THP. Based on our computational results, the following key conclusions can be drawn:

First, the hydroxylation of γBB to L-carnitine proceeds via a classical three-step mechanism for Fe(II)/αKG-dependent dioxygenases: Fe^IV^=O isomerization, hydrogen atom abstraction, and hydroxyl rebound. The quintet state is the reactive ground state, with the hydrogen atom abstraction step being rate-determining, exhibiting an energy barrier of 15.2 kcal/mol.

Second, the inhibition mechanism of BBOX by THP is governed by the electronic properties of the substrate. Following hydrogen abstraction, N-N bond cleavage (5.6 kcal/mol) is kinetically preferred over hydroxyl rebound (20.7 kcal/mol). This divergence is attributed to the substitution of the carbon atom in γBB with a nitrogen atom in THP, demonstrating how a single heteroatom can redirect an enzymatic reaction pathway.

Third, a comprehensive evaluation of possible rearrangement pathways led to a significant refinement of the mechanism. The initially proposed direct 1,2-H migration was ruled out due to an excessively high energy barrier. A revised mechanism was proposed, featuring imine-mediated hydrogen transfer, hydroxyl rebound preceding C-C bond formation, and final radical coupling. In this pathway, the hydroxyl rebound step becomes the overall rate-determining step for the inhibition reaction, with an energy barrier of 23.5 kcal/mol.

This work provides a detailed atomic-level understanding of both the catalytic and inhibitory mechanisms of BBOX, revealing how substrate electronic effects dictate reaction outcomes. The elucidated reaction pathways and energy profiles offer a robust theoretical foundation for understanding the catalytic versatility of the αKG-dependent dioxygenase family. Furthermore, the detailed mechanistic insights, including the identification of key intermediates and rate-limiting steps, provide valuable structural and energetic guidance for the rational design of novel, potent BBOX inhibitors. The computational framework proposed here can be readily extended to study other members of this important enzyme family, such as TMLH and the JMJD family of histone demethylases, to achieve a more comprehensive understanding of their catalytic mechanisms and facilitate the development of selective modulators.

## Data Availability

Data are contained within the article.

## Supplementary Materials

The following supporting information can be downloaded at: https://www.mdpi.com/article/10.3390/molecules31111941/s1. Additional tables and figures (Tables S1–S3 and Figures S1–S12) are provided. Figure S1: Overlap of the QM/MM optimized ^5^R structures extracted at 15 ns (green), 17 ns (megenta), 20 ns (purple), 25 ns (cyan), and 30 ns (orange) during the 30-ns MD simulation; Figure S2: Comparison of optimized ^5^R and ^5^R_0_ structures obtained by representative snapshots from 30-ns and 500-ns MD simulations; Figure S3: Optimized structures of transition states and intermediates involved in the hydroxylation of γBB catalyzed by BBOX; Figure S4: Optimized structure of transition state of *pro-R* hydrogen (H2) abstraction by Fe^IV^=O; Figure S5: (a) Schematic diagram of the expanded QM region (including Arg366). (b) QM/MM optimized ^3^R_QM_ and ^5^R_QM_ structures using the expanded QM region. (c) Potential energy profile of the process from R_QM_ to IM1_QM_ at the quintet state using the expanded QM region; Figure S6: Optimized structures of transition states and intermediates involved in the N-N bond cleavage (a) and OH rebound (b) of IM1′; Figure S7: Optimized structures involved in three proposed pathways for 1,2-H shift; Figure S8: Scanned diagram for the process of shortening of C1⋯C2 distance starting with IM4′_A_; Figure S9: Optimized structures involved in C-C bond formation and successive hydrogen transfer in route B; Figure S10: RMSDs for the backbone atoms of BBOX enzyme-substrate complex in 30 ns MD simulations based on the CHARMM all-atom force field; Figure S11: RMSDs for the backbone atoms of BBOX enzyme-substrate complex in 500 ns MD simulations based on the AMBER force field; Figure S12: Statistical analysis of the distance between H1 of the substrate and O atom of Fe^IV^=O over time in the 500ns MD simulation; Table S1: Energies (a.u.) of all local minima and transition states along the THP-induced oxidative rearrangement pathway at the quintet state; Table S2: Spin densities of key atoms/groups of all local minima and transition states along the THP-induced oxidative rearrangement pathway at the quintet state; Table S3: Imaginary Frequencies (cm^−1^) analysis of key transition states in the quintet calculated on the QM region.
