# Supplementary material for: Mechanistic Elucidation of BBOX-Catalyzed Hydroxylation and THP-Induced Oxidative Rearrangement via QM/MM Calculations
Source: Molecules. 2026 Jun 3;31(11):1941. doi: 10.3390/molecules31111941 (PMC13257696; doi:10.3390/molecules31111941)
Supplement: Supplementary file 1 [file molecules-31-01941-s001.zip › molecules-4268868-supplementary.pdf]

Mechanistic Elucidation of BBOX-Catalyzed Hydroxylation and THP-Induced Oxidative Rearrangement via QM/MM Calculations

Zheng Ruan, Hong Li, Yongjun Liu, Xianghui Zhang and Xinyi Li

Table S1. Energies (a.u.) of all local minima and transition states along the THP-induced oxidative rearrangement pathway at the quintet state.

Species	E_{opt}	E_{sp}	E_{DFT-D3}	E_{sp+D3}
**R′**	−4369.663432	−4370.152208	−0.287500	−4370.439708
**TS1′$_{rotate}$**	−4369.648910	−4370.132415	−0.285142	−4370.417557
**R′$_{rotate}$**	−4369.671250	−4370.162548	−0.288315	−4370.450863
**TS1′**	−4369.661085	−4370.149470	−0.287210	−4370.436680
**IM1′**	−4369.678938	−4370.173948	−0.289027	−4370.462975
**TS2′$_{H3}$**	−4369.673111	−4370.165494	−0.288556	−4370.454050
**IM2′$_{H3}$**	−4369.689244	−4370.191921	−0.290177	−4370.482098
**TS3′$_{C}$**	−4369.681550	−4370.179775	−0.289415	−4370.469190
**IM3′$_{C}$**	−4369.683779	−4370.183172	−0.289683	−4370.472855
**TS4′$_{C}$**	−4369.660336	−4370.148270	−0.287135	−4370.435405
**IM4′$_{C}$**	−4369.689125	−4370.191776	−0.290162	−4370.481938
**TS5′$_{C}$**	−4369.680224	−4370.176273	−0.289254	−4370.465527
**IM5′$_{C}$**	−4369.683312	−4370.182588	−0.289629	−4370.472217
**TS6′$_{C}$**	−4369.674022	−4370.163470	−0.288668	−4370.452138
**IM6′$_{C}$**	−4369.676483	−4370.167181	−0.288941	−4370.456122

**TS7′$_C$**	−4369.660011	−4370.147671	−0.287097	−4370.434768
**P′**	−4369.684006	−4370.184259	−0.289711	−4370.473970

Table S2. Spin densities of key atoms/groups of all local minima and transition states along the THP-induced oxidative rearrangement pathway at the quintet state.

Species	Fe	O	substrate	C1	N1
**R′**	3.13	0.58	0.00	0.00	0.00
**TS1′$_{rotate}$**	3.10	0.65	-0.02	0.00	0.00
**R′$_{rotate}$**	3.11	0.63	-0.01	0.00	0.00
**TS1′**	3.65	0.45	-0.50	-0.48	-0.01
**IM1′**	4.10	0.33	-0.93	-0.73	-0.01
**TS2′$_{H3}$**	4.50	0.15	-0.95	-0.14	-0.59
**IM2′$_{H3}$**	4.75	0.05	-0.98	-0.01	-0.67
**TS3′$_C$**	4.70	0.03	-0.90	-0.02	-0.55
**IM3′$_C$**	4.66	0.02	-0.85	-0.02	-0.25
**TS4′$_C$**	4.13	0.02	-0.46	-0.01	0.12
**IM4′$_C$**	3.68	0.01	0.27	-0.01	0.24
**TS5′$_C$**	3.79	0.01	0.19	-0.01	0.20
**IM5′$_C$**	3.97	0.01	0.12	-0.01	0.08
**TS6′$_C$**	3.91	0.00	0.13	0.55	-0.17
**IM6′$_C$**	3.89	0.00	0.14	0.67	-0.21
**TS7′$_C$**	3.90	0.00	0.06	0.01	-0.19
**P′**	3.95	0.00	0.04	0.01	0.03

Table S3. Imaginary Frequencies (cm^{-1}) analysis of key transition states in the quintet calculated on the QM region.

Species	TS_{rotate}	TS1	TS2	$TS1'_{rotate}$	TS1′	$TS2'_{H3}$	$TS3'_{C}$	$TS4'_{C}$	$TS5'_{C}$	$TS6'_{C}$	$TS7'_{C}$
Imaginary Frequency (cm^{-1})	-486.76	-966.49	-670.59	-466.55	-963.22	-564.54	-688.02	-1090.01	-862.20	-1178.79	-1067.84

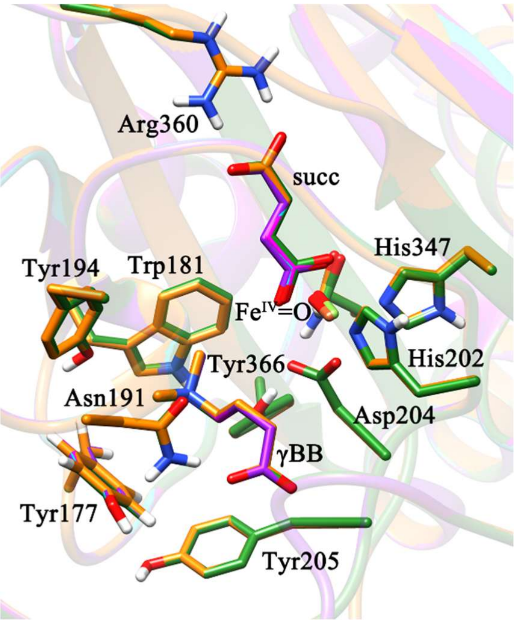

Figure S1. Overlap of the QM/MM optimized ^{5}R structures extracted at 15 ns (green), 17 ns (megenta), 20 ns (purple), 25 ns (cyan), and 30 ns (orange) during the 30-ns MD simulation.

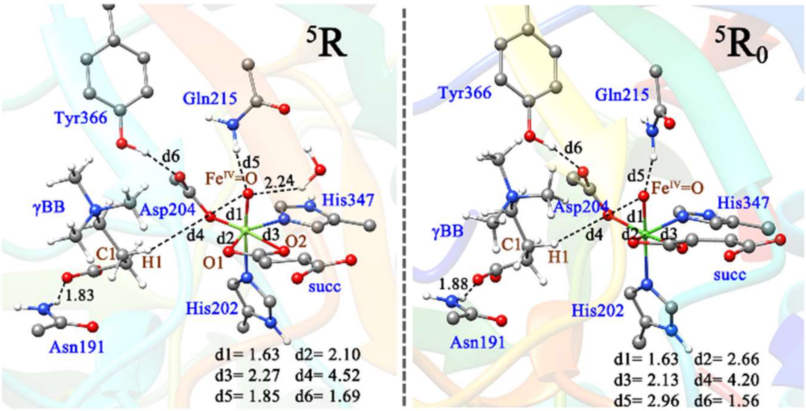

Figure S2. Comparison of optimized ^{5}R and ^{5}R$_0$ structures obtained by representative snapshots from 30-ns and 500-ns MD simulations, respectively.

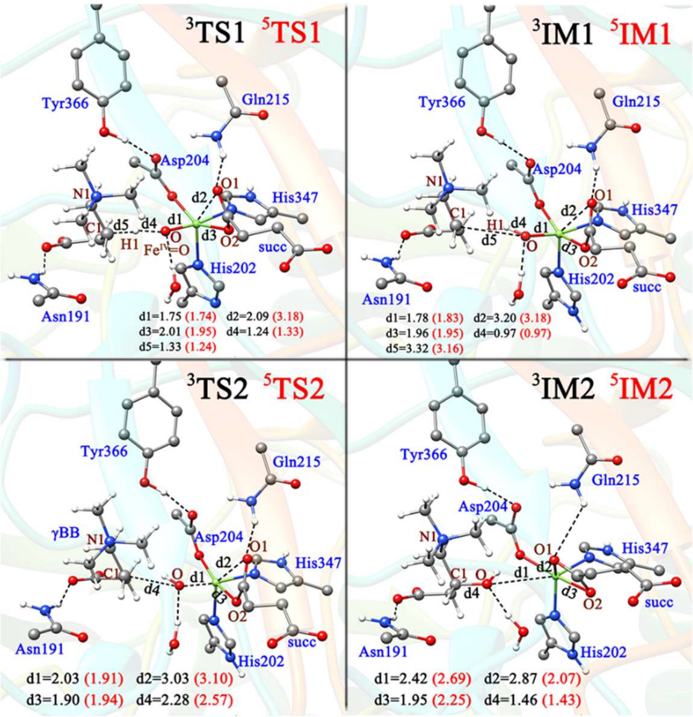

Figure S3. Optimized structures of transition states and intermediates involved in the hydroxylation of γBB catalyzed by BBOX. All distances are given in Ångström.

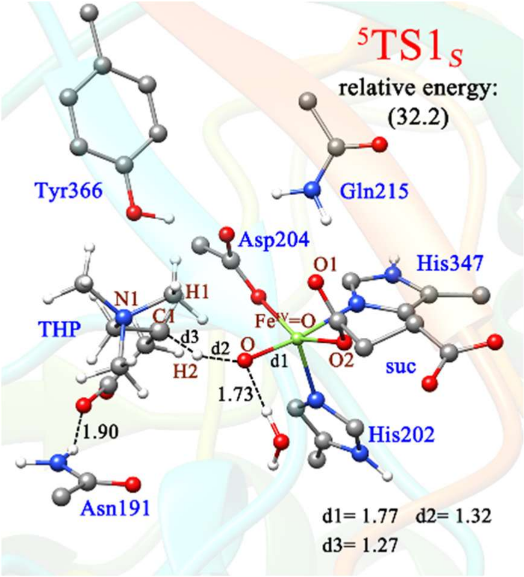

Figure S4. Optimized structure of transition state of *pro-R* hydrogen (H2) abstraction by Fe^{IV}=O. All distances are given in Ångström.

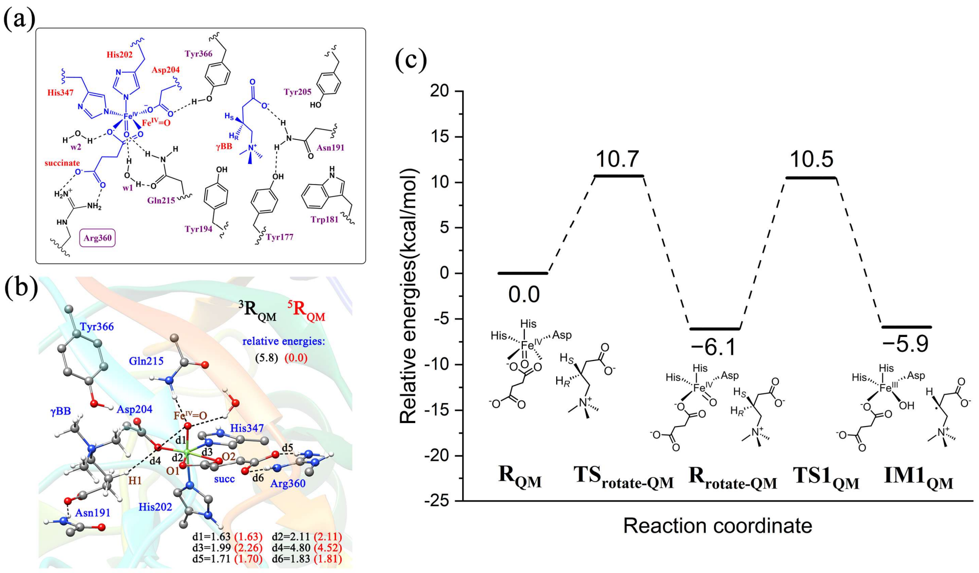

Figure S5. (a) Schematic diagram of the expanded QM region (including Arg366). (b) QM/MM optimized $^3R_{QM}$ and $^5R_{QM}$ structures using the expanded QM region. (c) Potential energy profile of the process from R_{QM} to $IM1_{QM}$ at the quintet state using the expanded QM region. All distances are given in Ångström.

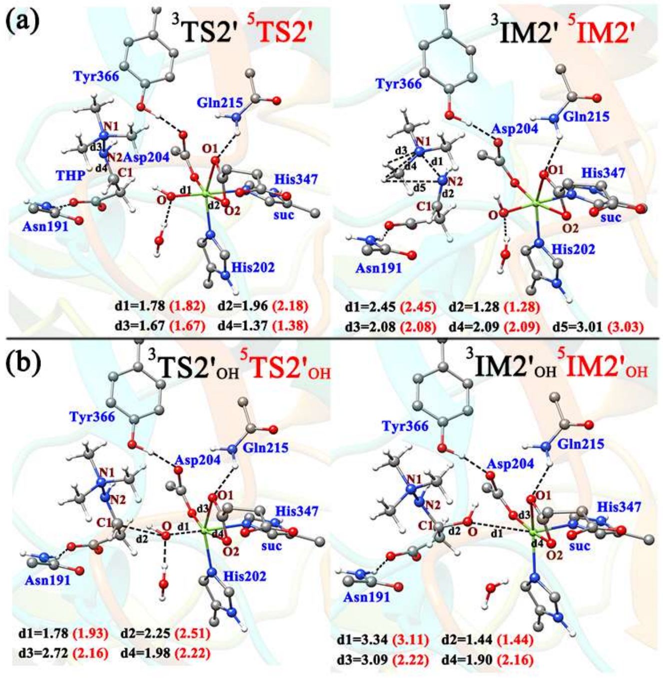

Figure S6. Optimized structures of transition states and intermediates involved in the N-N bond cleavage (a) and OH rebound (b) of IM1'. All distances are given in Ångström.

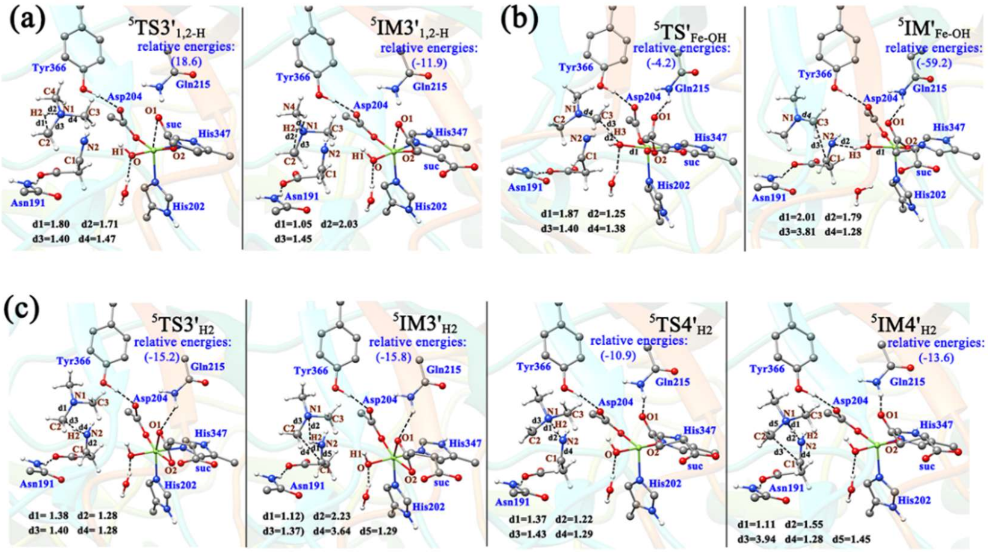

Figure S7. Optimized structures involved in three proposed pathways for 1,2-H shift. All distances are given in Ångström and relative energies are in kcal/mol.

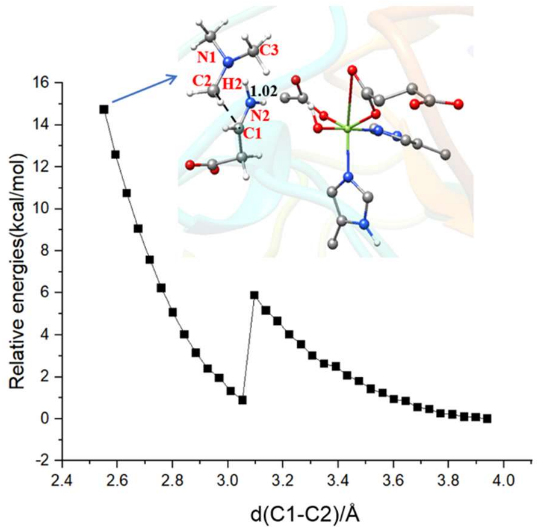

Figure S8. Scanned diagram for the process of shortening of C1···C2 distance starting with IM4'$_A$. Distances are given in Ångström.

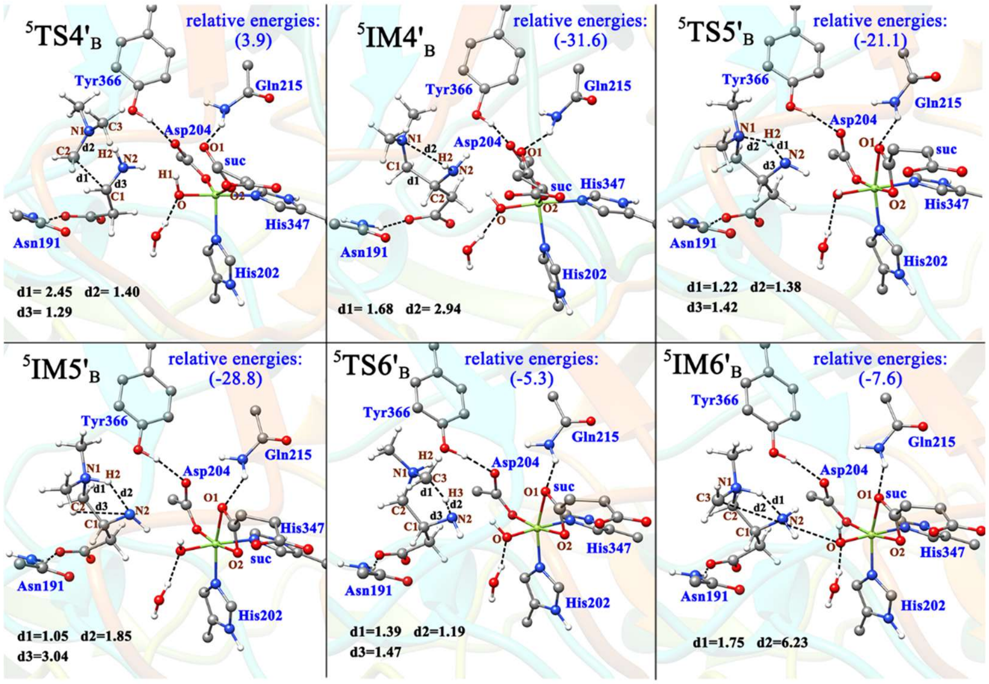

Figure S9. Optimized structures involved in C-C bond formation and successive hydrogen transfer in route B. All distances are given in Ångström and relative energies are in kcal/mol.

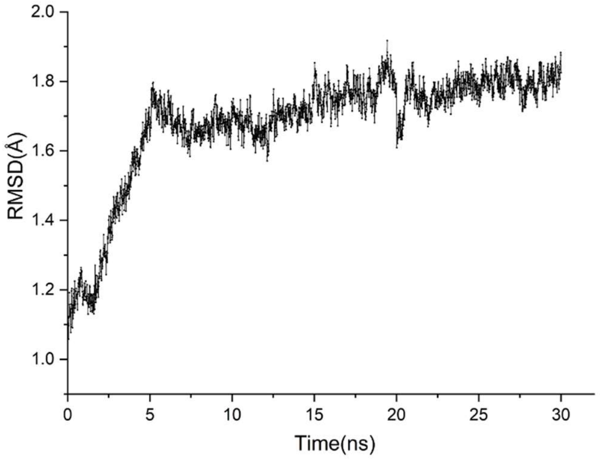

Figure S10. RMSDs for the backbone atoms of BBOX enzyme-substrate complex in 30 ns MD simulations based on the CHARMM all-atom force field.

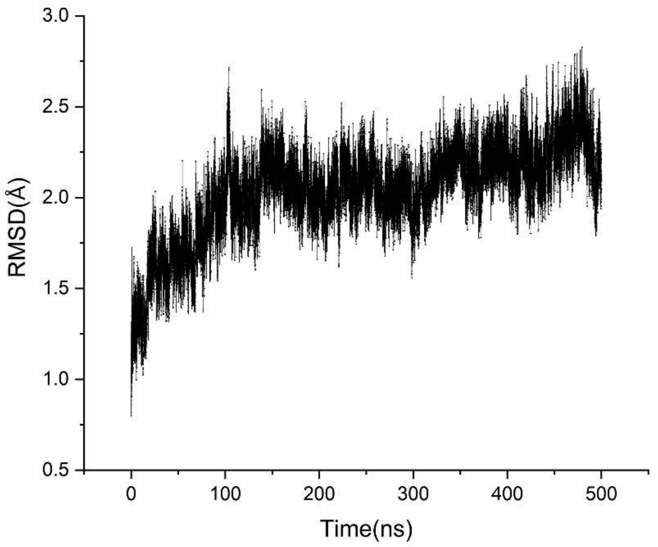

Figure S11. RMSDs for the backbone atoms of BBOX enzyme-substrate complex in 500 ns MD simulations based on the AMBER force field.

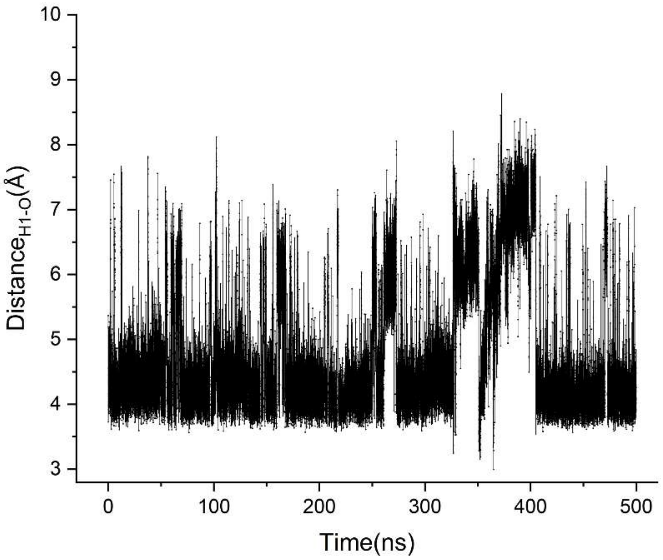

Figure S12. Statistical analysis of the distance between H1 of the substrate and O atom of Fe^{IV}=O over time in the 500ns MD simulation.
